# Carotid intima media thickness is associated with body fat abnormalities in HIV-infected patients

**DOI:** 10.1186/1471-2334-14-348

**Published:** 2014-06-23

**Authors:** Paula Freitas, Davide Carvalho, Ana Cristina Santos, António José Madureira, Esteban Martinez, Jorge Pereira, António Sarmento, José Luís Medina

**Affiliations:** 1 Endocrinology Department, Hospital de São João and University of Porto Medical School, Alameda Hernâni Monteiro, Porto 4200, Portugal; 2 Hygiene and Epidemiology Department, University of Porto Medical School, Porto, Portugal; 3 University of Porto Institute of Public Health, Porto, Portugal; 4 Radiology Department, Hospital de São João and University of Porto Medical School, Porto, Portugal; 5 Department of Infectious Diseases, Hospital Clinic, University of Barcelona Medical School, Barcelona, Spain; 6 Nuclear Medicine Department, Hospital de São João l, Porto, Portugal; 7 Infectious Disease Department, Hospital de São João and University of Porto Medical School, Porto, Portugal

**Keywords:** Lipodystrophy, HIV, Carotid intima media thickness, Fat mass ratio, Body composition

## Abstract

**Background:**

HIV-infected patients may be at increased risk of cardiovascular (CV) events, and lipodystrophy is generally associated with proatherogenic metabolic disturbances. Carotid intima-media thickness (cIMT) has been used as a surrogate marker for atherosclerosis and it has been shown to be an independent risk factor for CV disease. Our objective was to evaluate cIMT in HIV-infected patients on combined anti-retroviral therapy (cART) with and without lipodystrophy defined by fat mass ratio (L-FMR), and to determine the association of lipodystrophy and visceral obesity [(visceral (VAT), subcutaneous adipose tissue (SAT) volume and VAT/SAT ratio, objectively evaluated by CT scan] with cIMT.

**Methods:**

Cross-sectional study of 199 HIV-infected patients. Body composition by DXA and abdominal CT, lipids, blood pressure, inflammatory markers, and cIMT by ultrasonography were performed. L-FMR was defined as the ratio of the percentage of trunk fat mass to the percentage of lower limb fat mass by DXA. Categorical variables were compared using the chi-square or Fisher’s exact test. Spearman correlation coefficients were estimated to study the association between cIMT and clinical and metabolic characteristics. Means of cIMT, adjusted for age, were calculated, using generalized linear models.

**Results:**

L-FMR was present in 41.2% of patients and cIMT was higher in these patients [0.81 (0.24) vs. 0.76 (0.25); p = 0.037)]. Lipodystrophic patients had higher VAT and VAT/SAT ratio and lower SAT. cIMT was associated with lipodystrophy evaluated by FMR, trunk fat, total abdominal fat, VAT and VAT/SAT ratio. No association was observed between cIMT and leg fat mass. Using generalized linear models, cIMT means were adjusted for age and no significant differences remained after this adjustment. The adjusted mean of cIMT was 0.787 (95% CI: 0.751-0.823) in patients without lipodystrophy, and 0.775 (95% CI: 0.732-0.817) in those with lipodystrophy (p = 0.671).

**Conclusions:**

HIV-infected patients on cART with lipodystrophy defined by FMR, had a significantly higher cIMT. Carotid IMT was also associated with classical cardiovascular risk factors. In these patients, visceral adipose tissue had a significant impact on cIMT, although age was the strongest associated factor.

## Background

The development of atherosclerosis lesions is a slow and progressive process. The use of noninvasive surrogate markers of atherosclerosis may improve the prognostic stratification of HIV-infected patients with cardiovascular risk factors, and also clarify some of the pending issues related to this disease in HIV infection. Ultrasound is a safe, inexpensive and rapid technique, which allows an accurate evaluation of atherosclerosis [1]. Intima-media thickness (IMT) of the carotid arteries has been used as a surrogate marker for atherosclerosis and it has been shown to be an independent risk factor for cardiovascular disease. The role of age, prolonged survival, traditional and non-traditional risk factors, direct effect of HIV infection and cART, in relation to surrogate markers of atherosclerosis are under continuous debate [1]. Thus, due to the long-term survival associated with cART, harmful effects of the treatment emerge, as well as a chronic inflammatory state. However, HIV itself may also promote atherosclerosis by activating the vascular endothelium directly, or indirectly, by systemic cytokine stimulation by the virus [2]. Higher rates of sub-clinical atherosclerosis are seen in HIV-1-infected patients, and, controversially, these are usually attributed to classic cardiovascular risk factors and to the side effects of cART [3-5].

Although the disease can cause alterations in several metabolic pathways, such as lipid and glucose metabolism, lipodystrophic patients, *per se,* may also be at a higher risk of atherosclerosis, as fat redistribution is associated with the presence of several known metabolic risk factors for cardiovascular diseases [6]. Furthermore, patients with clinical lipodystrophy have a significantly higher risk of coronary heart disease at 10 years, measured by the Framingham risk score, than patients without clinical lipodystrophy and those with clinical lipodystrophy and metabolic syndrome were more often classified in moderate and high risk categories, than those without metabolic syndrome [7].

The objectives of this study were to evaluate cIMT in HIV-infected patients subject to cART, according to the presence of lipodystrophy, as objectively defined by FMR, and also to evaluate the association of lipodystrophy, visceral obesity [(visceral (VAT), subcutaneous adipose tissue volume (SAT) and VAT/SAT ratio objectively evaluated by CT scan] and other established cardiovascular risk factors, with cIMT.

## Methods

### Subjects

As part of a cross-sectional study, clinically stable, HIV-infected, non-institutionalized Caucasian adults who were receiving antiretroviral therapy were evaluated. These were all referred from the Infectious Diseases Outpatient Clinic and only patients subject to cART were included. This study was approved by the Ethics Committee for Health of the Hospital São João in Porto and each patient agreed to provide written, informed consent.

### Clinical assessment

For each patient, the following information was collected, using a standardized protocol: age, gender, known duration of HIV infection and duration of cART exposure, HIV risk factor, characterization of the infection, smoking history (past, current, or never), family history of CV diseases, and use of anti-hypertensive, anti-diabetic, or lipid lowering drugs.

We used the “Centers for Disease Control and Prevention” (CDC) HIV staging classification [8]. Weight, height, circumferences of neck, waist, hip, thigh and arm were measured, as previously described [9]. All measurements were performed by the same observer, using standard techniques [10].

Resting blood pressure (BP) taken whilst in a supine position was measured in a standardized fashion, as previously described [7].

### Evaluation of body composition

Body composition was assessed with whole-body, dual-energy X-ray absorptiometry (DXA) (DXA – Lunar Expert XL, 1999). DXA measurements were performed while the patient was in a supine position, with standard positioning of the arms and feet. Markers for the trunk and lower limbs that defined regions of interest were defined in accordance with the manufacturer’s instructions. Regional fat mass values were grouped and analyzed for the following anatomical regions: arms, legs, trunk and total body. The fat mass ratio (FMR) was calculated as the ratio between the percentage of trunk fat mass and the percentage of lower limb fat mass (FMR =% of the trunk fat mass/% of the lower limb fat mass) [11]. We used a cut-off value for lipodystrophy defined by FMR for men of 1.961, and 1.329 for women [12].

The quantification of total, visceral, and subcutaneous fat was performed with a 64-slice, abdominal computed tomography (CT) scanner (Siemens Sensation 64 Cardiac), with the same technique as previously described [13,14]. All values were expressed in cm^2^, rounded to the nearest centesimal.

### Laboratory analysis

Biological and inflammatory parameters:

A venous blood sample was taken after a 12-hour overnight fast. All the samples were analyzed at the central laboratory of our hospital. The measurements of total cholesterol (TC), low density lipoprotein (LDL) cholesterol, high-density lipoprotein (HDL) cholesterol, triglycerides, apolipoprotein A1 (apo A1), apolipoprotein B (apo B), lipoprotein (a) [Lp (a)], fibrinogen, high sensitivity C-reactive protein (hsCRP), homocysteine, uric acid, lactates, NT-proBNP, glucose, insulin and A1c serum levels were determined using commercial kits. Non HDL-C was defined as TC-HDL. Microalbuminuria was determined in a 24 hour urine sample. Patients without a previous diagnosis of diabetes were submitted to an oral glucose tolerance test (OGTT). This test was performed as instructed by the World Health Organization [15].

The CD4+ cell count (×10^6^ cell/L) was determined by flow cytometry and plasma HIV-1 RNA loads were measured by a quantitative reverse transcriptase polymerase chain reaction (Roche Diagnostic Systems, Inc., Branchburg, NJ, USA), which had a lower limit of detection of 50 copies/mL.

### Measurements of insulin resistance

Insulin resistance was defined by the homeostasis model assessment of insulin resistance (HOMA), using the following formula:

**HOMA ‒ IR index **= (insulin 0 x glucose 0)/22.5 [16].

### Carotid IMT measurement

High-resolution B-mode and Doppler ultrasonography of the carotid arteries was carried out with a Philips iU22 machine (Philips Medical, The Netherlands), equipped with a 17–5 MHz high-frequency linear-array transducer. Patients were examined in the supine position, with the head in a neutral position, or slightly turned away from the side that was being scanned. The left and right common carotid arteries (CCA) were examined in multiple directions and measurements were performed using the images with the best wall definition. IMT was measured 3 cm below the carotid bulb in a longitudinal image. Three measurements were obtained on each side, and the mean IMT of these values was calculated. All studies were performed by the same radiologist, who had 12 years of experience in vascular ultrasound, and were carried out using the same machine.

The presence of subclinical carotid atherosclerosis was defined as IMT > 0.80 mm (70th percentile of the IMT distribution), presence of plaque, or both [17-19].

### Statistical analysis

Data was described as mean and standard deviation (SD) for quantitative variables, and was compared using Student-t or Mann–Whitney tests, as appropriate. Categorical variables were described as counts and proportions, and compared using the chi-square or Fisher’s exact test. To study the association between cIMT and clinical and metabolic characteristics, Spearman correlation coefficients were estimated. Means of cIMT, adjusted for age, were calculated, using generalized linear models.

Statistical analysis was performed using SPSS version 17.0 software (SPSS Inc., Chicago, Illinois, USA). All probabilities were two tailed and p values of <0.05 were regarded as significant.

## Results

### Patient characteristics

In this sample of 199 HIV-1 infected patients (132 men and 67 women) on cART, 41.2% presented lipodystrophy defined by FMR. Table 1 shows the characteristics of the study sample, according to the presence of lipodystrophy defined by FMR.

**Table 1:** Sample characteristics according to the presence of lipodystrophy defined by FMR

	**Without L**	**With L**	***P***
**n (%)**	**117**	**82**	**-**
Gender [n (%)]			
Male	71 (60.7)	61 (74.4)	0.063
Female	46 (39.3)	21 (25.6)	
Age [years, mean (sd)]	44.8 (12.1)	49.2 (10.2)	0.002
Duration of HIV infection [years, mean (sd)]	7.2 (3.8)	8.5 (3.5)	0.005
cART [years, mean (sd)]	5.7 (3.7)	7.9 (3.3)	<0.001
Weight [Kg, mean (sd)]	68.0 (14.3)	68.0 (10.6)	0.805
Height [m, mean (sd)]	1.64 (0.09)	1.64 (0.08)	0.518
BMI [(Kg/m^2^), mean (sd)]	25.2 (5.0)	25.3 (4.0)	0.637
Waist circumference [cm, mean (sd)]	90.6 (12.7)	92.6 (10.2)	0.174
Hip circumference [cm, mean (sd)]	96.1 (9.4)	92.5 (6.4)	0.005
Thigh circumference [cm, mean (sd)]	48.5 (5.5)	47.0 (4.9)	0.025
Arm circumference [cm, mean (sd)]	26.7 (3.2)	27.2 (2.6)	0.285
Neck circumference [cm, mean (sd)]	36.6 (3.8)	37.8 (3.8)	0.045
Waist/hip circumference ratio [mean (sd)]	0.94 (0.09)	1.00 (0.07)	<0.001
CD4 cell count [cells/mm3, mean (sd)]	502.7 (265.0)	631.8 (340.8)	0.006
HIV RNA (<50) [n (%)]	102 (87.2)	74 (90.2)	0.660
HIV risk factor [n (%)]			
Injecting drug user	32 (27.8)	15 (18.3)	0.050
Homosexual contact	7 (6.1)	14 (17.1)	
Heterosexual contact	74 (64.3)	51 (62.2)	
Others	2 (1.7)	2 (2.4)	
CDC [n (%)]			
A	63 (54.3)	53 (64.6)	0.209
B	1 (0.9)	0 (0)	
C	52 (44.8)	29 (35.4)	
ART [n (%)]			
PI	59 (51.3)	48 (58.5)	0.390
NNRTI	58 (50.4)	38 (46.3)	0.673
NRTI	112 (97.4)	79 (96.3)	0.695
Smoking history [n (%)]			
Never	44 (37.9)	36 (43.9)	
Current	55 (47.4)	27 (32.9)	
Former	17 (14.7)	19 (23.2)	0.092
Familial history of CVD [n (%)]	51 (43.6)	40 (48.8)	0.563
Taking medications [n (%)]			
Statins	22 (19.0)	25 (30.5)	0.088
Fibrates	39 (33.6)	39 (47.6)	0.067
Oral anti-diabetics	13 (11.2)	16 (19.5)	0.154
Insulin	6 (5.2)	4 (4.9)	1.000
Anti-hypertensive drugs	21 (17.9)	19 (23.5)	0.442
Fat mass [%, mean (sd)] DXA			
Total	24.8 (13.1)	19.3 (8.5)	0.003
Trunk	25.7 (13.3)	24.3 (9.0)	0.446
Leg	24.4 (14.7)	11.1 (7.4)	<0.001
Arm	27.2 (17.1)	20.3 (13.6)	0.007
Fat mass [Kg, mean (sd)] DXA			
Total	17.3 (11.1)	13.2 (7.3)	0.013
Trunk	9.3 (6.0)	9.0 (4.8)	0.918
Leg	5.2 (3.6)	2.1 (1.6)	<0.001
Arm	2.1 (1.9)	1.5 (1.2)	0.019
Body fat mass by quantitative CT			
Total fat [cm^2^, mean (sd)]	282.2 (174.5)	276.6 (124.0)	0.865
VAT [cm^2^, mean (sd)]	111.5 (85.8)	158.2 (63.2)	<0.001
SAT [cm^2^, mean (sd)]	170.8 (125.7)	118.3 (93.1)	0.002
VAT/SAT ratio [cm^2^, mean (sd)]	1.08 (1.46)	2.43 (2.62)	<0.001
Systolic blood pressure [mmHg, mean (sd)]	120.0 (18.9)	124.6 (15.8)	0.039
Diastolic blood pressure [mmHg, mean (sd)]	75.8 (11.9)	78.0 (10.6)	0.139
Leukocytes [10^9^/L, mean (sd)]	5.66 (1.67)	6.39 (1.88)	0.007
Glucose 0 min [mg/dL, mean (sd)]	104.4 (45.1)	114.7 (44.2)	0.003
Glucose 2 hours [mg/dL, mean (sd)]	123.6 (42.9)	139.2 (50.3)	0.051
Insulin 0 min [μU/mL, mean (sd)]	9,49 (10.28)	13.00 (10.68)	0.002
Insulin 2 hours [μU/mL, mean (sd)]	52.43 (46.65)	91.32 (147,24)	0.007
HOMA [mean (sd)]	2,54 (2.90)	3.73 (3.31)	<0.001
A1c [% mean (sd)]	5.50 (1.06)	5.77 (0.06)	0.001
Total cholesterol [mg/dL, mean (sd)]	218.6 (53.2)	233.6 (61.0)	0.093
LDL-cholesterol [mg/dL, mean (sd)]	126.1 (47.1)	132.3 (50.7)	0.461
HDL-cholesterol [mg/dL, mean (sd)]	47.0 (14.4)	44.3 (12.0)	0.262
Non-HDL cholesterol [mg/dL, mean (sd)]	171.6 (48.9)	189.3 (56.5)	0.019
Triglycerides[mg/dL, mean (sd)]	247.5 (170.9)	307.2 (198.5)	0.006
Apo A1 [mg/dL, mean (sd)]	118.8 (25.8)	114.3 (18.3)	0.204
Apo B [mg/dL, mean (sd)]	104.7 (26.7)	106.1 (25.4)	0.827
Ratio apo B/apo A1 [mean (sd)]	0.91 (0.27)	0.95 (0.25)	0.297
Lp (a) [mg/dL, mean (sd)]	30.2 (31.2)	31.1 (39.7)	0.421
Homocysteine [μmol/L, mean (sd)]	9.6 (4.1)	9.8 (3.7)	0.435
CRP [mg/L, mean (sd)]	5.4 (12.9)	4.0 (4.2)	0.913
hsCRP [mg/dL, mean (sd)]	0.48 (0.91)	0.37 (0.28)	0.185
Lactates [mmol/L, mean (sd)]	1.12 (0.47)	1.36 (0.58)	0.001
NT-ProBNP [pg/mL, mean (sd)]	39.1 (67.3)	28.6 (27.1)	0.414
Fibrinogen [mg/dL, mean (sd)]	344.8 (98.3)	336.2 (92.1)	0.463
Microalbumin [mg/L, mean (sd)]	49.1 (150.6)	26.0 (67.7)	0.435
Uric acid [mg/L, mean (sd)]	44.4 (15.2)	51.2 (15.4)	<0.001
Carotid IMT [mm, mean (sd)]	0.76 (0.25)	0.81 (0.24)	0.037

(L – Lipodystrophy FMR defined; CDC – Centers for Disease Control and Prevention criteria for staging of HIV infection; cART – combined antiretroviral therapy; BMI – body mass index; FMR – fat mass ratio; CVD – cardiovascular disease; DXA – dual-energy X-ray absorptiometry; CT – computed tomography; PI – protease inhibitor; NNRTI – non-nucleoside reverse transcriptase inhibitor; NRTI – nucleoside reverse transcriptase inhibitor, VAT – visceral adipose tissue, SAT – subcutaneous adipose tissue, IMT – intima-media thickness).

Patients with lipodystrophy were older, had been infected with HIV for a longer time and had a greater length of cART. No differences in weight, height, BMI, waist and arm circumferences among patients with, or without lipodystrophy, were observed. Patients with lipodystrophy had lower hip and thigh circumferences, but higher neck circumference and a higher waist/hip circumference ratio than those without lipodystrophy. Furthermore, patients with lipodystrophy had a higher systolic blood pressure and leukocyte count and a mean CD4+ cell count.

No differences were observed between patients with, and without, lipodystrophy regarding viral suppression rate, HIV risk factor, CDC classification and ART regimens, smoking history, family history of CV disease and medication history (statins, fibrates, oral anti-diabetics, insulin and anti-hypertension drugs).

### Body composition by DXA

In the evaluation of body composition by DXA, patients with lipodystrophy had a lower total, leg and arm fat mass, both in terms of % and Kg. No difference was observed in trunk fat mass (in % and Kg) between the two groups.

Patients with lipodystrophy had a higher VAT and VAT/SAT ratio, lower SAT and no significant difference in total fat at abdominal level.

### Metabolic parameters

Patients with lipodystrophy had higher glucose and insulin at 0 minutes, and insulin at 120 minutes on OGTT, HOMA, non-HDL cholesterol, triglycerides, uric acid, and lactates, than patients without lipodystrophy. No significant differences were found in the 2-hour value of glucose on OGTT, total cholesterol, LDL-cholesterol, HDL-cholesterol, apo A1, apo B, ratio Apo B/Apo A1, Lp(a), homocysteine, CRP, hsCRP, NT-ProBNP, fibrinogen and microalbumin urinary excretion between the two groups.

### Carotid IMT measurements

**Figure 1:**
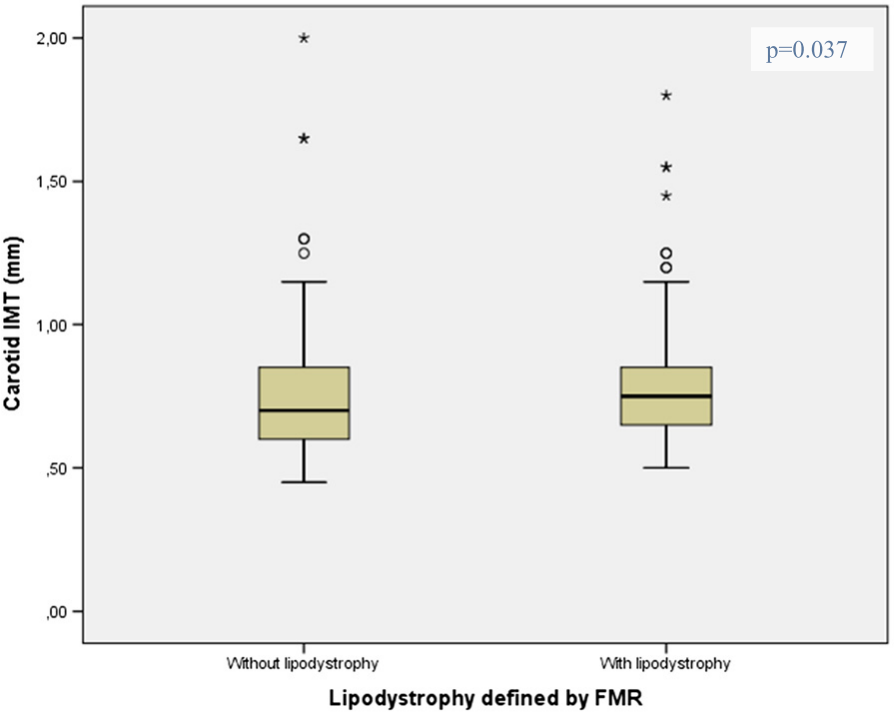
Mean differences of carotid IMT by the presence of lipodystrophy defined by FMR.

Carotid IMT was higher in patients with lipodystrophy, than in patients without lipodystrophy [mean (SD) 0.81 (0.24) vs. 0.76 (0.25); p = 0.037)] (Figure 1).

Carotid IMT correlated positively with lipodystrophy evaluated by FMR, age, waist/hip ratio, neck circumference, trunk fat mass evaluated by DXA, visceral obesity defined by total abdominal fat, VAT and VAT/SAT ratio, systolic blood pressure, glucose at 0 and 120 minutes on OGTT, A1c, non-HDL cholesterol, uric acid, CRP, hsCRP and homocysteine. No significant correlations were found between IMT and duration of HIV infection, length of cART, thigh circumference, total, leg and arm fat mass evaluated by DXA, CD4 cell count, leukocyte, insulin at 0 and 120 minutes on OGTT, HOMA, triglycerides, and lactates (Table 2).

**Table 2:** Correlations between cIMT and body composition and metabolic parameters

**Correlations between cIMT and**	**R**	**P**
Age	0.709	<0.001
Duration of HIV infection	−0.019	0.779
cART	0.024	0.727
Waist/hip ratio	0.387	<0.001
Thigh circumference	−0.095	0.169
Neck circumference	0.202	0.007
CD4 cell count	−0.027	0.701
Systolic blood pressure	0.419	<0.001
Leukocytes	−0.049	0.478
Glucose 0 min	0.284	<0.001
Glucose 2 hours OGTT	0.220	0.006
Insulin 0 min	0.026	0.711
Insulin 2 hours OGTT	−0.089	0.271
HOMA	0.094	0.185
A1c	0.285	<0.001
Triglycerides	0.109	0.113
Non-HDL cholesterol	0.254	<0.001
CRP	0.196	0.005
hsCRP	0.334	0.003
Homocysteine	0.150	0.032
Uric acid	0.146	0.034
Lactates	0.081	0.253
Fat mass total	0.084	0.236
Trunk	0.168	0.018
Leg	−0.037	0.604
Arm	0.029	0.686
Total fat on abdominal CT scan	0.218	0.002
VAT on abdominal CT scan	0.418	<0.001
SAT on abdominal CT scan	−0.009	0.901
VAT/SAT ratio on abdominal CT scan	0.290	<0.001
FMR	0.250	p < 0.001

(IMT – intima-media thickness; cART – combined antiretroviral therapy; CDC – Centers for Disease Control and Prevention criteria for staging of HIV infection; cART – combined antiretroviral therapy; OGTT – oral glucose tolerance test; HOMA – the homeostasis model assessment of insulin resistance; HDL-high density cholesterol; CPR –C protein reactive; VAT – visceral adipose tissue; SAT – subcutaneous adipose tissue; CT – computed tomography; FMR – fat mass ratio).

p = 0.037.

Using generalized linear models, cIMT means were adjusted for age, and no significant differences remained after this adjustment when patients with, and without, lipodystrophy were compared. The age adjusted mean of cIMT was 0.787 (95% CI: 0.751-0.823) in patients without lipodystrophy, and 0.775 (95% CI: 0.732-0.817) in patients with lipodystrophy (p = 0.671).

## Discussion

Our aim was to evaluate the association between objectively defined lipodystrophy and cIMT. Prior studies that had evaluated the effect of lipodystrophy on cIMT, had conflicting results [20-22]. We found that cIMT was higher in patients with lipodystrophy defined by FMR, than in patients without lipodystrophy [0.81 (0.24) vs. 0.76 (0.25); p = 0.037)]. Coll et al. showed that the presence of lipodystrophy defined by clinical parameters increased by 3-fold the risk of sub-clinical carotid atherosclerosis in patients with HIV-infection [21]. On the other hand, Mercié et al. showed a significant association between cIMT and lipodystrophy in a univariate analysis, but the effect disappeared in the multivariate model [20]. Coll et al. suggested that this discrepancy of results was due to the younger and healthier population of the latter, since the mean cIMT was 0.54 mm (range 0.50-0.60), while in the Coll’s samples, the mean cIMT was 0.80 (range 0.40-1.80). In Coll’s study, the mean age of the patients with lipodystrophy was 42.38 (1.09) years while in Mercié’s it was 43.7 (8.3) years. The mean age of Coll’s patients without lipodystrophy was 40.59 (0.75), while in the Mercié et al’s study, it was 39.4 (8.7) years. Our patients, either with lipodystrophy [49.2 (10.2) years], or without lipodystrophy [44.8 (12.1) years], were older than those of these previous published studies. Despite being older than those of Coll et al., our patients had a mean cIMT identical to Coll’s patients [with lipodystrophy cIMT 0.88 (0.04) vs. without lipodystrophy 0.76 (0.02) mm; p = 0.007], but similar to Mercié’s study, no significant differences remained after adjustment for age.

Visceral obesity is another independent risk factor for CVD in the background population [23] and independent associations of obesity and abdominal adiposity with cIMT were observed [24]. We also found that cIMT was positively correlated with waist/hip ratio, neck circumference, and trunk fat mass evaluated by DXA and visceral obesity defined by total abdominal fat, VAT and VAT/SAT ratio by CT scan. However, the mechanisms by which obesity is linked to early carotid atherosclerosis are not clearly established, even though the role of metabolic factors, such as insulin resistance and altered plasma adiponectin levels, has been proposed. Yet body composition and fat distribution may influence systemic hemodynamics - both systemic blood pressure and total blood volume [25].

Insulin resistance has been independently associated with vascular disease and accelerated CV disease in HIV-infected adults [26]. In our sample, patients with lipodystrophy had higher glucose and insulin at 0 and 120 minutes on OGTT, and greater HOMA than patients without lipodystrophy. Carotid IMT correlated positively with glucose at 0 and at 120 minutes on OGTT and A1c. Despite the higher insulin resistance evaluated by HOMA, no significant correlations were observed between cIMT and HOMA.

Some studies have demonstrated that traditional CV risk factors overshadow the role of HIV infection and/or cART, and that they are the major determinants of cIMT progression [4,27-30]. Sankatsing et al. have shown that HIV infection and/or cART were associated with increased cIMT independent of traditional CV risk factors [31], and Lekakis et al. pointed to the combination of all parameters [32]. Older age is a traditional CV risk factor, associated with higher mean cIMT and a higher prevalence of carotid lesions [30,33]. We also found that cIMT correlated positively with age. As with Mercié’s study, cIMT was significantly associated with age and also with waist- to-hip ratio, systolic BP, non-HDL cholesterol, glucose disorders and homocysteine.

Adverse lipid and metabolic changes associated with HIV infection or cART exposure may be important pro-inflammatory factors [34], and plasma lipid alterations have been associated with an increased cIMT [35]. We found that cIMT was positively correlated with non-HDL cholesterol and was also positively correlated with systolic blood pressure. Similar to Mercié et al., we also observed an association between total plasma homocysteine and cIMT in HIV-infected patients subject to cART, as was previously demonstrated in non-infected patients [20].

It should be noted that certain “classical” vascular risk factors are over-represented in the HIV-infected population, e.g. smoking. However, we observed no differences between patients with, and without, lipodystrophy, with regards to smoking history. Also, no differences were observed between patients with, and without, lipodystrophy, with regards to viral suppression rate, HIV risk factor, CDC classification and ART regimens, family history of cardiovascular disease, and also medications taken (oral anti-diabetic drugs, insulin and anti-hypertension drugs). Statins are known to have beneficial effects on the arterial wall [36], but, once again, no differences were observed with those taking statins and fibrates.

In HIV-negative patients, both leukocyte count and CRP and hsCRP have been linked to endothelial dysfunction and future CV events [37,38]. Similar to Hsue, we found that hsCRP was associated with cIMT. However, in Hsue’s study, after adjusting for all traditional risk factors, the association between hsCRP and cIMT was no longer significant [39].

### Study limitations

The limitations of this study are mainly related to the observational design and the cross-sectional nature of our analyses, which preclude any conclusions regarding causality. Subclinical atherosclerosis evaluated by carotid IMT, reflects the cumulative effects of risk factors acting over many years, whereas we measured risk factors at only one point in time. Thus, blood lipid concentrations, blood pressure, smoking habits and other CV risks estimated at one point in time, may not accurately represent lifetime exposure. On the other hand, we only used a qualitative assessment of smoking habits and not a quantitative one. Moreover, cART, CD4 + T-cell count, HIV viral load, and CVD risk mediators are dynamic variables which change over time.

Some aspects of our study need to be highlighted. Firstly, we only used objective methods for the evaluation of body composition: the presence of lipodystrophy was defined by FMR by DXA and visceral obesity by a CT scan. Secondly, this is one of the first reports in which cIMT was used as an outcome. And thirdly, we emphasize that increased cIMT could be a predictor of clinical cardiovascular endpoints, namely, coronary and cerebrovascular ones [18].

## Conclusions

HIV-infected patients under cART with FMR defined lipodystrophy had a significantly higher cIMT when compared with patients without lipodystrophy. Carotid IMT in a univariate analyses was positively associated with lipodystrophy defined by FMR, VAT, VAT/SAT ratio, age and other classical cardiovascular risk factors. Visceral adipose tissue had an impact on cIMT in HIV-patients subject to cART, but no significant associations remained when this was adjusted for age.

## Competing interests

The authors declare that they have no competing interests.

## Authors’ contributions

PF conceived the study, participated in its design, in the acquisition of data, and drafted the manuscript; DC conceived the study, participated in its design, and drafted the manuscript; ACS performed the statistical analysis and critically revised the manuscript; AJM performed the CT scans and reviewed the data; JP performed DXA scans and reviewed the data; EM and AS critically revised the manuscript; JLM revised the study design. All authors read and approved the final manuscript.

## Pre-publication history

The pre-publication history for this paper can be accessed here:

http://www.biomedcentral.com/1471-2334/14/348/prepub
